# Ehlers–Danlos Syndrome Type Arthrochalasia: A Systematic Review

**DOI:** 10.3390/ijerph19031870

**Published:** 2022-02-07

**Authors:** Marta Martín-Martín, Jonathan Cortés-Martín, Maria Isabel Tovar-Gálvez, Juan Carlos Sánchez-García, Lourdes Díaz-Rodríguez, Raquel Rodríguez-Blanque

**Affiliations:** 1 Virgen de las Nieves University Hospital, 18014 Granada, Spain; martamartinm97@gmail.com; 2 Research Group CTS1068, Andalusia Research Plan, Junta de Andalucía, 18014 Granada, Spain; jonathan.cortes.martin@gmail.com (J.C.-M.); matoga@ugr.es (M.I.T.-G.); cldiaz@ugr.es (L.D.-R.); raquel.rodriguez.blanque.sspa@juntadeandalucia.es (R.R.-B.); 3 Department of Nursing, School of Health Sciences, University of Granada, 18016 Granada, Spain; 4 Department of Nursing, School of Health Sciences, Ceuta Campus, University of Granada, 51001 Ceuta, Spain; 5 San Cecilio University Hospital, 18016 Granada, Spain

**Keywords:** Ehlers–Danlos syndrome (EDS), EDS arthrochalasia, rare disease, connective tissue, congenital anomaly, hypermobility, systematic review

## Abstract

Ehlers–Danlos syndrome type arthrochalasia (aEDS) is a rare genetic disease characterized by severe generalized joint hypermobility, bilateral congenital hip dislocation, skin hyperextensibility, muscle hypotonia, and mild dysmorphic features. It is an autosomal dominant connective tissue disease causing defects in collagen, associated with two genes, COL1A1 or COL1A2. Only about 42 cases have been published worldwide. Treatment is currently symptomatic and focuses on increasing the quality of life of these patients, as there is no curative treatment. The main objective of the review was to update information on Ehlers–Danlos syndrome type arthrochalasia from scientific publications. The review report was carried out in accordance with the criteria of the Preferred Reporting Items for Systematic reviews and MetaAnalyses (PRISMA) review protocol, by searching Orphanet, OMIM, PubMed, and Scopus, as well as free sources. A total of 20 articles were analyzed, which, after analysis, provide an updated report that aims to establish a solid starting point for future lines of research.

## 1. Introduction

Ehlers–Danlos syndrome (EDS) is a rare disease, with a genetic origin which is one of the rare connective tissue diseases that cause alterations in collagen. The disease has a number of subtypes that share a clinical basis, but there are differences in the development of the disease [1]. Some of the shared clinical manifestations among the different types of EDS are soft and hyperextensible skin, abnormal scars, hematomas, and hypermobility of articulations. Other clinical features may also be present, depending on the type of EDS, such as the cardiac-valvular type with progressive aortic and mitral valve problems or the periodontal type with severe periodontitis [2].

Thirteen different types of EDS, caused by the alteration of 19 genes, have been identified. A 2017 classification was updated with the description of another genetically distinct type, provisionally classified as classic EDS type 2, which brought the total number of associated genes to 20 [2]. Most types of EDS have a known genetic cause resulting from pathogenic variants in genes, but there are also patients who have clinical features that are compatible with EDS but do not fit into a currently defined type. This indicates that the genetic heterogeneity of EDS has not been fully resolved [3]. Studies conducted in 2002 estimate the incidence to be 1:5000 [4], although the prevalence of the different types of EDS is currently unknown, so research is vital.

The clinical manifestations are very diverse between the different types of EDS and between patients with the same type. Clinical manifestations can occur in all systems of the body [5]. The observation of the presence of such manifestations should guide towards the initial diagnosis, followed by a study of the family history and collagen alterations. In the diagnosis of vascular and arthrochalasia subtypes, a test on skin fibroblast samples is made to detect collagen alterations and to measure the activity of the decreased enzymes due to the mutation of the specific gene [6].

The diagnosis of EDS is no easy matter as many of its clinical manifestations can be found in other diseases or even in the general population. This makes the differential diagnosis complicated as there are many conditions that must be considered. Among such conditions are (1) Hereditary connective tissue disorders that predispose to aneurysm formation and blood vessel fragility such as Loeys–Dietz syndrome, Marfan syndrome, and familial thoracic aortopathy conditions; (2) Diseases presenting with hematomas and predisposition to nonaccidental injuries or abnormal skin, including coagulation disorders, pseudoxanthoma elasticum, and cutis laxa; (3) Neuromuscular conditions related to collagen VI and XI disorders, such as Ullrich congenital muscular dystrophy and Bethlem myopathy; (4) Connective tissue disorders that present with a degree of joint hypermobility, such as skeletal dysplasias, osteogenesis imperfecta, chondrodysplasias, and Larsen syndrome; (5) Other specific genetic conditions such as Stickler syndrome, Noonan syndrome, and mucopolysaccharidosis. This complex differential diagnosis results in a situation where EDS is often not diagnosed or confirmed until advanced stages of the disease [6].

There is currently no specific treatment that will change the course of EDS [5]. The only available treatments are symptomatic and will vary between patients as it depends on how the syndrome affects the person. Diagnosed individuals should be treated by a multidisciplinary team and receive multidisciplinary treatment. Life expectancy depends on the specific type of syndrome; it is shortened in vascular type EDS due to rupture of organs and vessels and may also be shortened in kyphoscoliosis type. In the other types, life expectancy is usually not inferior to that of the general population [7].

After a brief description of the disease, the literature review will focus on a specific subtype, EDS arthrochalasia (aEDS). AEDS is caused by heterozygous mutations resulting in partial or complete loss of exon 6 of the COL1A1 (17q21.33) or COL1A2 (7q21.3) genes causing bilateral congenital hip dislocation, generalized joint hypermobility, and hyperextensible and/or fragile skin [8]. A better understanding of the disease would hopefully shorten the time to diagnosis and thus provide better comprehensive care for each patient.

## 2. Materials and Methods

### 2.1. Review Protocol

The methodology used to prepare this report was a systematic review of the scientific literature published on aEDS, following the review protocol Preferred Reporting Items for Systematic reviews and Meta-Analyses (PRISMA), which consists of a 27-point checklist of the most representative sections of an original article, as well as a flow chart describing the information search process. This systematic review was carried out following a protocol registered on the PROSPERO website and whose registration number is CRD42021233501.

### 2.2. Eligibility Criteria

We selected original studies that provided information on aEDS, with no restrictions on the language of publication, published between 2011 and 2021. Any type of article or review was accepted. Due to the paucity of documented cases, the literature review will focus on a specific subtype, EDS arthrochalasia.

### 2.3. Sources of Information and Search Strategy

The literature search was performed in the Orphanet [8], OMIM (Online Mendelian Inheritance in Man) [9], PubMed, and Scopus databases. A manual search was performed using reference lists of studies to find other relevant studies. The structured language used was obtained using MeSH terms and Health Sciences (DeCS) descriptors. The descriptors used were “Ehlers–Danlos syndrome”, “arthrochalasia”, and the Boolean operator used was “AND”, together with reference # 130060 in OMIM [9] and ORPHA1899 in ORPHANET [8].

The search strings used are shown in Table 1.

### 2.4. Data Mining Process

The articles found were transferred to the Mendeley web application using the Mendeley web importer tool. After exporting all the articles to the Mendeley website, they were organized into folders according to the database from which they had been obtained, and these in turn were organized into sub-folders by subject. Finally, all duplicates were eliminated, leaving the definitive study list.

### 2.5. Selection of Studies

In order to select the articles that could be related to the data we intended to collect, a selective reading of the title and abstract of all the studies found was carried out. Each article was then read individually, focusing on the methodology to check whether it met the inclusion criteria, discarding documents in which no relationship was found with the objectives and characteristics of this review.

### 2.6. Synthesis of Results

Based on the information provided by this review, we obtained a series of premises that will serve to support further studies on this subject, in addition to a complete analysis of the pathology.

## 3. Results

**Figure 1:**
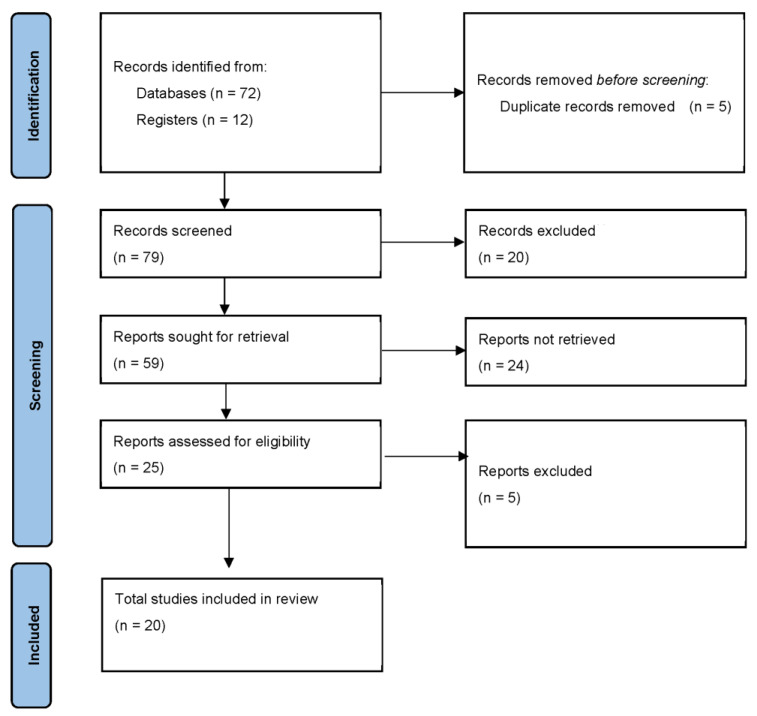
Flow chart.

Figure 1 describes the flow chart of the articles used for this literature review.

After a thorough reading of the 25 selected articles, five articles based exclusively on case reports of other types of EDS were excluded.

The objectives and results of the studies in this review are presented in Table 2.

The results of the selected studies are shown below.

Conducting studies on the evolution and description of this type of disease is a difficult task due to the low prevalence, the geographical dispersion of cases, and the paucity of documented comprehensive follow-ups of affected individuals. Numerous authors have provided information through their research on cases of patients diagnosed over time, such as Hatamochi et al., who documented the first case in Japan [17].

Ayoub et al. [20], Brady et al. [18], Hakim et al. [13], and Giunta et al. [1] describe aEDS as a rare autosomal dominant connective tissue disorder, which is characterized by clinical manifestations such as congenital bilateral hip dislocation, severe generalized hypermobility of articulations, recurrent articular dislocations and subluxations, and hyperextensibility of the dermis.

In addition to the characteristic clinical manifestations of the disease, less prevalent manifestations are observed in the cases described. The most notable are those cited by Ayoub et al. [20], such as muscular hypotonia, kyphoscoliosis, radiologically mild osteopenia, tissue fragility, including atrophic scarring, and hematomas.

Rolfes et al. [19], in their research on whether EDS causes increased bone fragility during infancy and childhood, did not identify an increased incidence of fractures in children with aEDS. In addition, in the study by Basalom et al. [22], they observed a very low incidence of fractures in adults diagnosed with this type of syndrome.

The description of the disease continues to grow as it is directly related to the emergence of new cases. Studies such as that of Melis et al. [16] describe a case of aEDS in which progressive valvular involvement is identified. No other similar cases have been described in the literature as they did not receive cardiological follow-up, so cardiac follow-up is warranted for patients with aEDS.

With regards to pregnancy, there are studies of pregnant women affected by aEDS, in which pregnancy or delivery-related complications including breech presentation, polyhydramnios, premature rupture of membranes, and decreased fetal movement have been observed [18]. Women affected by EDS should be followed closely during pregnancy.

Only one case of exitus has been reported, of a baby born at 35 gestational weeks diagnosed with aEDS post-mortem, who presented with a skull fracture and bilateral hip dislocations with metaphyseal sclerosis after an uncomplicated vaginal delivery. Genetic testing revealed a pathogenic variant in the COL1A1 gene with exon 6 skipping not inherited from either parent [15].

The diagnosis of aEDS becomes highly important in the neonatal period considering the potential consequences of physical disabilities in the adult years. Newborns diagnosed in the literature with aEDS have distinctive features of the syndrome, including mild hypertelorism, bilateral epicanthal folds, large fontanels, and especially pronounced micrognathia. Another difference between aEDS and other types is that neonatal hypotonia is frequently severe. Hypotonia seems to decrease with age along with generalized hypermobility. Mental development of all patients is normal [15].

The early diagnosis of this type of disease is currently very complex. Studies such as Brady et al. [18] and Liu et al. [21] describe multiple differential diagnoses such as Larsen’s syndrome, Loeys–Dietz syndrome, or cutis laxa, which make it difficult to reach a definitive early diagnosis.

Malfait et al. [2] indicate that molecular testing is essential to achieve a definitive diagnosis of aEDS. Early molecular testing will help in the treatment of the disease’s signs and/or symptoms.

Analyzing genetic studies, all authors indicate that aEDS is caused by heterozygous mutations in COL1A1 or COL1A2 leading to the loss of exon 6 in either gene during pre-mRNA processing, thus impairing the proper formation of collagen fibrils. COL1A1 and COL1A2 encode the pro-α1 and pro-α2 chains of type I collagen protein. In the past, this syndrome underwent a variation in terminology: aEDS was subdivided into type VIIA if the α1 (I) chain was affected or VIIB if the α2 (I) chain was affected [10].

Several studies, such as those by Brady et al. [18] and Byers et al. [10], suggest that COL1A1-associated aEDS may be more severe than COL1A2-associated aEDS.

The results of the studies reviewed indicate that there is still no curative treatment for aEDS [20].

## 4. Discussion

### 4.1. Summary of Evidence

A general description of the aEDS type is given below. It can be found in the OMIM (Mendelian Inheritance in Man) project database under reference # 130060 [9] and in ORPHANET under reference ORPHA1899 [8].

#### 4.1.1. Epidemiology

AEDS is a rare disorder with an unknown prevalence [18]. To date, about 42 patients have been reported worldwide [17].

#### 4.1.2. Etiology

AEDS is a rare autosomal dominant connective tissue disorder characterized by severe generalized joint hypermobility with multiple dislocations including bilateral congenital dislocation of the hips, muscle hypotonia, mild dysmorphic features, and skin abnormalities [15].

Throughout history, aEDS has been referred to as “arthrochalasis multiplex congenita” or “Ehlers–Danlos syndrome type VII (VIIA, VIIB)” [18].

#### 4.1.3. Pathophysiology

AEDS is caused by heterozygous mutations in COL1A1 or COL1A2 that result in partial or complete translocation of exon 6 in either of those mutations during pre-mRNA conversion. COL1A1 and COL1A2 both encode the pro-α1 and pro-α2 strands of type I collagen protein with a triple helix structure composed of two α1 strands and one α2 strand. Type 1 procollagen molecules deposited in the extracellular space are transformed into mature type 1 collagen molecules, a principal component of the ligaments, tendons, dermis, bone, and dentine, by proteolytic cleavage of N- and C-propeptides. Partial or complete deletion of exon 6 in COL1A1 or COL1A2 gives rise to partial or complete depletion of the *N*-telopeptide linking the *N*-propeptide to the main triple helix domain. The loss of the segmental *N*-telopeptide for the pro-α1 and pro-α2 chains causes the removal of the small globular region of the *N*-propeptide (present only in the pro 1α chain), the scission region of the procollagen *N*-proteinase, the cross-linking lysine residual of the *N*-telopeptide, and the main helical domain first triplet Gly-X-Y, which results in the retention of the *N*-propeptide in the mature α1(I) and α2(I) molecules. This undermines the appropriate formation of collagen fibrils [18].

#### 4.1.4. Clinical Manifestations

The hallmarks of aEDS are severe generalized hypermobility of articulations, bilateral congenital hip dislocation, and/or recurrent subluxations and dislocations of small and large articulations, skin hyperextensibility, and muscle hypotonia.

Patients with aEDS tend to have dysmorphic characteristics that are also among the distinguishing characteristics of this syndrome. These characteristics include mild hypertelorism, bilateral epicanthal folds, large fontanelles, and especially pronounced micrognathia [15].

#### 4.1.5. Diagnosis

The aEDS has several major diagnostic criteria and minor diagnostic criteria (Table 3).

This tool facilitates the possible diagnosis of aEDS based on the manifestations that appear [20].

The minimum criteria for diagnosis are the presence of a combination of congenital bilateral hip dislocation with cutaneous hyperextensibility or with generalized joint hypermobility, with at least two other minor criteria [20].

The final diagnosis of aEDS is made by molecular testing. Genetic testing is performed when there is a strong clinical suspicion and it is justified regardless of age [6].

Diagnosis is important in the neonatal period because of the potential consequences of physical disability in adulthood [15].

#### 4.1.6. Differential Diagnosis

In particular, this type of disease can lead to differential diagnoses such as Larsen’s syndrome due to the joint dislocations it causes, classic EDS, dermatosparaxis EDS, kyphoscoliotic EDS, musculocontractural EDS due to the multiple clinical manifestations they have in common, Loeys–Dietz syndrome, and cutis laxa, as they are all hereditary connective tissue disorders [18].

#### 4.1.7. Treatment

There is currently no specific curative treatment for aEDS. The only available treatment is symptomatic, and it can vary greatly depending on how the syndrome affects the individual. At the time of diagnosis, a whole-body skeletal survey is advisable to aid treatment [20].

Early prenatal or postnatal diagnosis can be life-saving, and early appropriate intervention can alleviate the physical and psychological suffering of the patient. Invasive surgery should be omitted in this type of patient and will be particularly unsuccessful in patients with aEDS [15]. The care of patients with aEDS focuses on the management of orthopedic problems with the primary aim of achieving stable deambulation. Open reductions with iliac osteotomy, with or without femoral osteotomy, are useful for the treatment of congenital hip dislocations. Certain guidelines should be taken into account to avoid complications such as leaving sutures twice as long as normal before suture removal and that wounds should be closed without tension [14].

With regards to the joints, stability can be improved with muscle-strengthening exercises, but weight-bearing exercises should be avoided. Patients with hyperextensibility should be educated about the range of extension they can perform in order to not exceed it. Joint protection and fall prevention are advised. Contact sports are discouraged [18].

Anti-inflammatory drugs can help patients with joint pain. In patients with muscle hypotonia and joint instability with chronic pain, emotional support and behavioral and psychological therapy to aid in accepting and offering coping strategies to manage disability is advised. Patients with long-term chronic pain may need support from mental health services [5].

Patients diagnosed with this disease should have regular check-ups that address all bodily systems to prevent further complications [18].

#### 4.1.8. Forecast

The life expectancy of patients with aEDS is generally not inferior to that of the general population [5].

Although the clinical characteristics of the patients imply significant difficulties in participating in activities of daily living, we consider it important to investigate the quality of life of these patients in order to explore what can be done to improve it.

### 4.2. Limitations

EDS is a disease that has several clinical subtypes and multiple overlapping clinical manifestations, variability in presentation, and other unique features. These elements limit the possibilities of reaching a rapid and accurate diagnosis. Each patient diagnosed with EDS presents a unique clinical course over time, so there is no common pattern to help predict prognosis. Early diagnosis is a goal to be achieved in order to improve the quality of life of patients through early interventions.

There are major limitations to the development and research of this disease due to the low prevalence as well as the inexistence of comprehensive follow-ups of the documented cases.

Research into this disease is also affected by geographical dispersion, the time gap between cases, and the multiple differential diagnoses.

Scientific studies have given way for great advances in this disease such as the identification of the genetic origin of some types of EDS. However, definitive genetic tests are only available for two forms of EDS, one of them being aEDS, so it is very important to focus future studies on the genetic heterogeneity of this disease in order to establish an effective diagnosis for all types.

### 4.3. Possible Lines of Research

Both in this disease and in many others whose treatment is still unknown, the aim of treatment is often based on improving the quality of life of its patients. To this end, a plan of specific interventions with a multidisciplinary approach is desirable.

It is essential to review the cases documented in the literature and new cases in order to increase the sample of patients diagnosed with EDS in order to carry out a study to promote knowledge of the disease and contribute to making an early diagnosis more likely.

Of particular note is the need for standardization and universalization of the comprehensive assessment and diagnostic tests necessary for the diagnosis and management of the disease.

## 5. Conclusions

The main features of aEDS are generalized joint hypermobility, bilateral congenital hip dislocation and/or recurrent subluxations and dislocations of small and large joints, and skin hyperextensibility. Knowledge of the disease allows for early management of the disease, reducing the impact on the patient’s life. A comprehensive, multidisciplinary approach is vital for treatment and prevention of complications.

Encouraging the translation of EDS research findings into clinical practice is vital to facilitate early and to aid into the development of therapeutic alternatives.

Nurses are in a key position to recognize the signs and symptoms of EDS and thus can encourage an improvement in the quality of life of these patients through early interventions.

There is no strict treatment protocol, as it focuses on each patient’s specific needs in terms of symptomatology. Recommendations are centered on orthopedic management to ensure deambulation, pain control, maintenance, and improvement of quality of life and adjustment of the patient to the environment and activities of daily living.

Quality of life is usually diminished in all types of EDS due to the multiple complications that the syndrome entails in most cases.

## Figures and Tables

**Table 1:** Search string.

Sources of Information	Search String
ORPHANET	ORPHA:1899
OMIM	# 130060
SCOPUS	(ALL (ehlers-danlos AND syndrome) AND ALL (arthrochalasia)) AND DOCTYPE (ar OR re) AND PUBYEAR > 2009
PUBMED	Search: (Ehlers-Danlos syndrome) AND (arthrochalasia) Filters: Full text, from 2010–2020 (“ehlers danlos syndrome” [MeSH Terms] OR (“ehlers danlos” [All Fields] AND “syndrome” [All Fields]) OR “ehlers danlos syndrome” [All Fields] OR (“ehlers” [All Fields] AND “danlos” [All Fields] AND “syndrome” [All Fields]) OR “ehlers danlos syndrome” [AllFields]) AND “arthrochalasia” [All Fields].

**Table 2:** Characteristics of the studies.

Author	Year	Article	Objectives	Results
Byers et al. [10]	1997	EDS Type VIIA and VIIB Result from Splice-Junction Mutations or Genomic Deletions That Involve Exon 6 in the COL1A1 and COL1A2 Genes of Type I Collagen.	Identification of mutations in EDS type VII.	EDS type VII is the result of defects in the conversion of the Procollagen type I to collagen, as a consequence of mutations in the substrate (EDS type VIIA and VIIB) or in the protease itself (VIIC).
Beighton et al. [11]	1998	Ehlers–Danlos Syndromes: Revised Nosology, Villefranche, 1997.	Categorization of types of EDS.	Description of each type of EDS so far.
Nicholls et al. [12]	2000	Clinical phenotypes and molecular characterization of three patients with Ehlers–Danlos syndrome type VII.	Investigation of 3 cases of EDS VII showing clinical phenotypes and molecular characterization.	Ten different genomic mutations are detected in patients with arthrochalasia-type EDS and all have in common the skipping of exon 6 sequences.
Hakim et al. [13]	2006	Joint hypermobility and skin elasticity: the hereditary disorders of connective tissue	Study of hereditary connective tissue disorders (HDCT).	The clinical manifestations of HDCT are very varied so it is of great importance to have a correct diagnosis.
Yen et al. [4]	2006	Clinical Features of Ehlers–Danlos Syndrome.	Review of 16 case records of EDS cases during the study period 1997–2002.	All patients had skin hyperextensibility, joint hypermobility and tissue fragility. It shows prevalence of common features.
Giunta et al. [1]	2008	The Arthrochalasia Type of Ehlers–Danlos Syndrome (EDS VIIA and VIIB): The Diagnostic Value of Collagen Fibril Ultrastructure.	To explain aspects of the diagnosis of arthrochalasia-type EDS.	Description of characteristics of the different modes of inheritance and associated manifestations together with their diagnosis.
Whitaker et al. [14]	2009	Molecular genetic and clinical review of Ehlers Danlos Type VIIA: implications for management by the plastic surgeon in a multidisciplinary setting.	Literature review on EDS VIIA and surgical problems.	Exercise extreme caution and preoperative planning for any patient with EDS type VII as they suffer from effects on healing and scarring of the skin and the susceptibility to bruising and hemorrhagic complications.
Klaassens et al. [15]	2011	Ehlers–Danlos arthrochalasia type (VIIA-B)-expanding the phenotype: from prenatal life through adulthood.	Description of 7 patients with aEDS diagnosed from prenatal life to adulthood.	Importance of type EDS diagnosis arthrochalasia in the neonatal period.
Melis et al. [16]	2012	Cardiac valve disease: an unreported feature in Ehlers Danlos syndrome arthrochalasia type?	Description of a case with a confirmed diagnosis of aEDS with mitral valve regurgitation and aortic and tricuspid regurgitation.	First report of valvular heart involvement in aEDS. It is likely that this feature was not detected due to the limited follow-up of patients.
Hatamochi et al. [17]	2014	The first Japanese case of the arthrochalasia type of Ehlers–Danlos syndrome with COL1A2 gene mutation.	Presentation of the first aEDS diagnosis in Japan.	Presentation of the clinical characteristics and the diagnostic process. A mutation was observed, the skipping of exon 6 encoding the protease cleavage site at the amino-terminal end of the proα1 (I) or proα2 (I) chains of type I collagen.
Brady et al. [18]	2017	The Ehlers–Danlos Syndromes, Rare Types.	Summary of the current knowledge on EDS subtypes and highlight areas for future research.	Full description of each EDS subtype.
Malfait et al. [2]	2017	The 2017 International Classification of the Ehlers–Danlos Syndromes.	Description of new subtypes of EDS.	The International EDS Consortium proposes a revised EDS classification, which recognizes 13 subtypes.
D’hondt et al. [7]	2018	Vascular phenotypes in nonvascular subtypes of the Ehlers–Danlos syndrome: a systematic review.	Research on vascular complications in “non-vascular” EDS subtypes.	Minor vascular complications were reported in EDS types arthrochalasia and other “non-vascular” types.
Rolfes et al. [19]	2019	Fracture incidence in Ehlers–Danlos syndrome. A population based case-control study.	Investigate whether EDS causes increased bone fragility during infancy and childhood.	There is no evidence that babies with EDS are predisposed to more frequent fractures.
Hein et al. [5]	2019	Ehlers–Danlos Syndrome: It’s Not Your Normal Hoofbeats.	Presentation, diagnosis, and management of the EDS.	Description of signs and symptoms by system, diagnostic tests and criteria, health education, and prognosis
Ghali et al. [6]	2019	Ehlers–Danlos syndromes.	Information on types of EDS	Compilation of updated information on types of EDS.
Ayoub et al. [20]	2020	Clinical features, molecular results, and management of 12 individuals with the rare arthrochalasia Ehlers–Danlos syndrome.	Presentation of clinical features, molecular diagnosis, and treatment of 12 individuals with aEDS.	Full description of the diagnostic process, characteristics, and treatment of each case.
Liu et al. [21]	2020	Pathologic Skull Fracture in a Near-Term Neonate with Arthrochalasia Type Ehlers–Danlos Syndrome: A Case Report.	They present a case with a pathological skull fracture after childbirth compatible with aEDS.	It reinforces the importance of early diagnosis.
Basalom et al. [22]	2020	Bone Disease in Patients with Ehlers–Danlos Syndromes.	To summarize the bone findings, mainly bone mass and fracture risk, in the syndromes of EDS.	Bone mineral density varies widely among the different types of EDS.
Malfait et al. [3]	2020	The Ehlers–Danlos syndromes.	Overview of the disease, mutations, and manifestations.	Detailed explanation of the most relevant aspects of the EDS syndrome.

**Table 3:** Diagnostic criteria for aEDS.

Main Diagnostic Criteria	Minor Diagnostic Criteria
Congenital bilateral hip dislocations	Muscle hypotonia
Severe generalized joint hypermobility with subluxations	Kyphoscoliosis
Recurrent dislocations of small joints	Radiologically mild osteopenia
Recurrent dislocations of large joints	Tissue fragility including atrophic scars
Hyperextensibility of the skin	Skin prone to bruising

## Data Availability

Not applicable.
